# Towards On-Demand *E. coli*-Based Cell-Free Protein Synthesis of Tissue Plasminogen Activator

**DOI:** 10.3390/mps2020052

**Published:** 2019-06-21

**Authors:** Seung-Ook Yang, Gregory H. Nielsen, Kristen M. Wilding, Merideth A. Cooper, David W. Wood, Bradley C. Bundy

**Affiliations:** 1 Department of Chemical Engineering, Brigham Young University, Provo, UT 84602, USA; seungook81@gmail.com (S.-O.Y.); gregnielsen0@gmail.com (G.H.N.); kmwilding13@gmail.com (K.M.W.); 2 Department of Chemical and Biomolecular Engineering, Ohio State University, Columbus, OH 43210, USA; merideth.cooper@gmail.com (M.A.C.); wood.750@osu.edu (D.W.W.)

**Keywords:** cell-free protein synthesis, CFPS, tPa, tissue plasminogen activator, ischemic stroke

## Abstract

Stroke is the leading cause of death with over 5 million deaths worldwide each year. About 80% of strokes are ischemic strokes caused by blood clots. Tissue plasminogen activator (tPa) is the only FDA-approved drug to treat ischemic stroke with a wholesale price over $6000. tPa is now off patent although no biosimilar has been developed. The production of tPa is complicated by the 17 disulfide bonds that exist in correctly folded tPA. Here, we present an *Escherichia coli*-based cell-free protein synthesis platform for tPa expression and report conditions which resulted in the production of active tPa. While the activity is below that of commercially available tPa, this work demonstrates the potential of cell-free expression systems toward the production of future biosimilars. The *E. coli*-based cell-free system is increasingly becoming an attractive platform for low-cost biosimilar production due to recent developments which enable production from shelf-stable lyophilized reagents, the removal of endotoxins from the reagents to prevent the risk of endotoxic shock, and rapid on-demand production in hours.

## 1. Introduction

Pharmaceutical research and development over the past few decades has discovered treatments for a multitude of diseases including stroke [1], cancer [2] and heart disease [3]. Although these innovative treatments have provided much relief, there is still an economic barrier for many patients in need due to the high cost of drug treatments. An example is Tissue plasminogen activator (tPa) which is the only FDA-approved drug to treat ischemic stroke and costs over $6000 [4]. Ischemic stroke is responsible for ~80% of all strokes with strokes annually causing over 5 million deaths and disabling ~50% of the survivors [5]. Fortunately, tPa, stroke education, and access to medical care has greatly reduced the mortality rate in developed countries, while the mortality rate is significantly higher in developing countries where patients cannot afford tPa and have less access to medical education and care [5]. While tPa is now off patent, no generic or biosimilar has been developed. Biosimilars are biological products approved for usage based on their highly similar functions with FDA-approved biological products. Biosimilars are beneficial in terms of cost savings and increased patient accessibility. However, biosimilars do not reduce drug prices to the extent that generics of small-molecule drugs do. Biosimilars are only 10–30% less than the original drug price [6]. Even so, it is estimated that biosimilar introduction into the market will save the U.S. up to $378 billion over the next 20 years [7]. Thus, there is an unmet technological need to produce biosimilar therapeutics at extremely low costs to enable developing countries have access to life-saving therapeutics.

*Escherichia coli*-based cell-free protein synthesis (CFPS) is increasingly becoming an attractive platform as a future low-cost biosimilar production. Advantages of *E. coli*-based CFPS include (1) an open reaction environment for greater control over reaction conditions, (2) the ability to lyophilize reagents for shelf-stable storage and stockpiling, (3) on-demand rapid production in response to needs, (4) the ability to express proteins in an endotoxin-free environment, (5) scalability from the microliter to 100 liter scale, (6) very low cost reagents, (7) PEGylation optimization [8], and (7) the ability to automate the protein production process [9,10,11,12,13,14,15,16,17,18]. The production of active forms of multiple therapeutic proteins has been demonstrated in recent years, targeting a variety of diseases [19,20]. Indeed, a recent report demonstrated the *E. coli*-based CFPS production of a biosimilar to the FDA-approved crisantaspase [21]. Here, we further assess CFPS as a tool towards the production of biosimilars by demonstrating the production of a highly disulfide-bonded therapeutic protein, tPa.

tPA is a serine protease and functions by activating plasminogen to dissolve blood plasma proteins found in blood clots. tPa has a total of 35 cysteine residues which allow the formation of 17 disulfide bonds [22,23,24], and consists of 527 amino acids with five domains (such as Kringle I, Finger, EGF, thrombolytic Kringle II, and protease domains) [25]. Currently, tPA is produced in Chinese hamster ovary (CHO) cells and is post-translationally glycosylated [26]. Other eukaryotic cell expression techniques suffer due to the product not folding correctly, transport limitations, and hyperglycosylation [22,27,28,29]. Part of the difficulty in producing tPA is the 17 disulfide bonds required for the correctly folded protein. Attempts to produce tPA in periplasm of *E. coli* have suffered from rare codon usage, mis-folding, and loss of function [23,24]. An engineered *E. coli* (Origami B, DE3 strain), where two thioredoxins (TrxA and TrxC) and three glutaredoxins were oxidized, was used to express a full length of tPA. However, the expression yields and the specific activity were not reported [22]. Here, we demonstrate for the first time the expression of active tPA in an *E. coli*-based CFPS environment using a glutathione buffer and prokaryotic disulfide bond isomerase C (DsbC) [30]. tPA expression methods with CFPS, the specific activity of tPa, and demonstration of produced tPa’s ability to lyse a human blood clot is described.

## 2. Materials and Methods

### 2.1. Extract Preparation

The *E. coli* extract was prepared using BL21 Star^TM^ (DE3) *E. coli* strain purchased from Invitrogen (Carlsbad, CA, USA). This strain harbored pOFX-GroEL/ES and pET40-DsbC, key components to the process. A starting culture of this *E. coli* BL21 Star^TM^ (DE3) strain grew in 5 mL of LB media (supplemented with 100 μg/mL spectinomycin and kanamycin) overnight at 37 °C with shaking at 280 rpm. The culture was transferred to 100 mL of LB media (supplemented with 100 μg/mL spectinomycin and kanamycin) and grown until reaching an OD600 reading of 2.0. The 105 mL culture was then transferred to 1 L of media in a Tunair flask. Then, 1 mM of Isopropyl β-D-1-thiogalactopyranoside (IPTG) was added upon reaching 0.6 of OD600 to overexpress T7 RNA polymerase. Cells were harvested at the end of the exponential growth phase (OD600 of 1.2) using centrifugation at 6000 RCF for 10 min at 4 °C. Cells were washed with chilled Buffer A (10 mM Tris-acetate pH 8.2, 14 mM magnesium acetate, 60 mM potassium glutamate, and 1 mM dithiothreitol (DTT)) by centrifugation at 6000 RCF for 10 min at 4 °C. Cells were re-suspended with the buffer A (g of cell/mL of buffer A), and protein synthesis machinery was extracted using an EmulsiFlex French Press homogenizer (Avestin, Ottowa, ON, Canada) at 20,000 psi. The homogenized cells were centrifuged at 12,000 RCF for 30 min at 4 °C to clear the lysate. The supernatant was incubated in a shaking incubator for 30 min at 280 rpm and 37 °C. The extract was flash-frozen in liquid nitrogen for one min before being stored at −80 °C.

### 2.2. Cell-Free Protein Synthesis

Cell-free protein synthesis of tPA was carried out in 80–100 μL reactions housed in a 2.0-mL Eppendorf tube at 37 °C for 3 h, with PANOxSP as an energy source as reported previously [10]. Expression of tPa was templated using a pET-tPa plasmid which encodes the expression of human tPa under a T7 RNA polymerase promoter. Upon analysis, the codon sequence contained some rare codons based on *E. coli* usage frequencies, but was deemed suitable for initial tPa expression studies. Each reaction contained 25% reaction volume of the cell extract, 1.2 nM plasmid, 12 to 15 mM magnesium glutamate, 1 mM 1,4-Diaminobutane, 1.5 mM Spermidine, 33.3 mM phosphoenolpyruvate (PEP), 10 mM ammonium glutamate, 175 mM potassium glutamate, 2.7 mM potassium oxalate, 0.33 mM nicotinamide adenine dinucleotide (NAD), 0.27 mM coenzyme A (CoA), 1.2 mM ATP, 0.86 mM CTP, 0.86 mM GTP, 0.86 mM UTP, 0.17 mM folinic acid, and 2 mM of all 20 amino acids except glutamic acid. The cell extract had overexpressed GroEL/ES and DsbC as mentioned above. Reactions were supplemented with purified DsbC where specified. Additionally, a 5 mM glutathione buffer (GSSG:GSH = 4:1) was included in the CFPS reaction reagents to support disulfide bond formation.

### 2.3. Measuring Protein Concentration

Next, 5 μM of radiolabeled (U-14C) Leucine (PerkinElmer, Waltham, MA, USA) was added to the CFPS reaction. A volume of 3 μL of the CFPS reaction was spotted on each of the three separate pieces of Whatman 3MM chromatography paper and dried at 37 °C. Spotted papers were placed in a beaker on ice and covered with 5% (v/v) TCA at 4 °C to precipitate the proteins onto the filter paper for 15 min. The solution was exchanged with fresh TCA three times. Following the TCA washing steps, the papers were dried at 37 °C. The radioactivity of both TCA-precipitated and non-TCA-precipitated samples was measured using a LS6500 Multipurpose Scintillation Counter (Beckman Coulter, Brea, CA, USA). The fraction of incorporated leucine in washed and unwashed protein were used to determine the amount of protein synthesized. Soluble yields were determined by sample centrifugation at 4 °C and 17,000 × *g* for 15 min, followed by TCA-precipitation and scintillation counting of the supernatants.

### 2.4. tPa Purification

The expressed tPA was dialyzed in NET buffer (150 mM NaCl and 20 mM Tris-HCl, pH 6.7). The cell-free reaction that produced tPA was inserted into a dialysis tubing bag (Spectra/Por, Rancho Dominguez, CA, USA) with a molecular weight cut off of 6–8 kDa. The sample was immediately dialyzed against 300 mL NET buffer for 18 h at 4 °C with three buffer exchanges. Dialyzed samples were loaded directly onto the HisPurTM Ni-NTA spin column (Thermo Scientific, Rockford, IL, USA) that was pre-equilibrated with NET buffer containing 10 mM imidazole. The column was equilibrated with sample proteins for 30 min at 4 °C. The column was washed with a NET buffer containing 10 mM imidazole (1.2 mL). tPA was eluted with a NET buffer containing 500 mM imidazole (300 μL). Eluted samples were concentrated by dialysis in 500 mL of NET buffer containing 40% glycerol.

### 2.5. tPa Activity Assay

The SensoLyte AMC tPA activity assay kit was purchased from AnaSpec (Fremont, CA, USA). The reaction buffer, AMC substrate, and standard AMC (fluorescence) were provided in the kit. A standard curve was plotted with varying concentration of the standard AMC. Pure tPA was added to 1x reaction buffer that was provided to make a 50-μL enzyme solution. Then, 50 μL of the AMC substrate solution was added directly to the enzyme solution for the activity measurement. Fluorescence was monitored at excitation/emission = 354/442 nm.

### 2.6. Blood Clot Lysis Assay

Blood for the clot lysis assay was a donation from the BYU Student Health Center. Aliquots of the blood were coagulated in the incubator at 37 °C for three hours. Non-coagulated blood was removed using an aspirator. The weight of the blood was measured before the incubation with tPA enzyme solution (1.9, 0.43, and 0 μM) in 100 μL of water. After 16 h of incubation with the tPA enzyme solution, the non-coagulated part was removed by using the aspirator, and the weight of the blood was measured.

## 3. Results and Discussion

### 3.1. tPA Expression and Specific Activity

**Figure 1:**
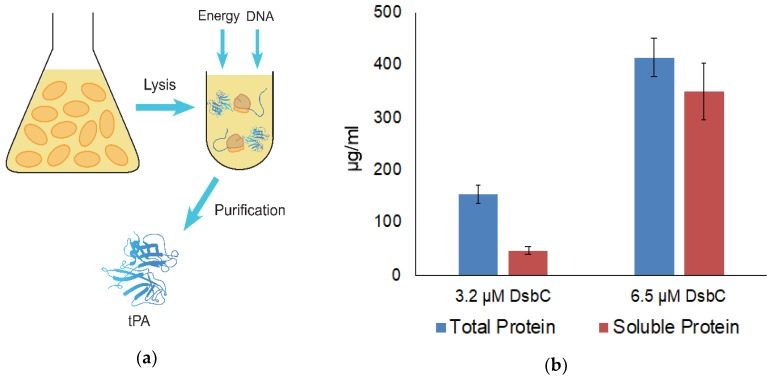
(**a**) Schematic showing the tissue plasminogen activator (tPA) expression process using a cell-free protein synthesis (CFPS) platform. *E. coli* cells are first grown and lysed. Next, cell extract, energy and DNA are all combined to express tPA which is then purified; (**b**) Total and soluble tPA yield with disulfide bond isomerase C (DsbC). Both reactions were incubated at 37 °C for 3 h. The final concentration of DsbC in each reaction is shown. Error bars represent one standard deviation.

**Figure 2:**
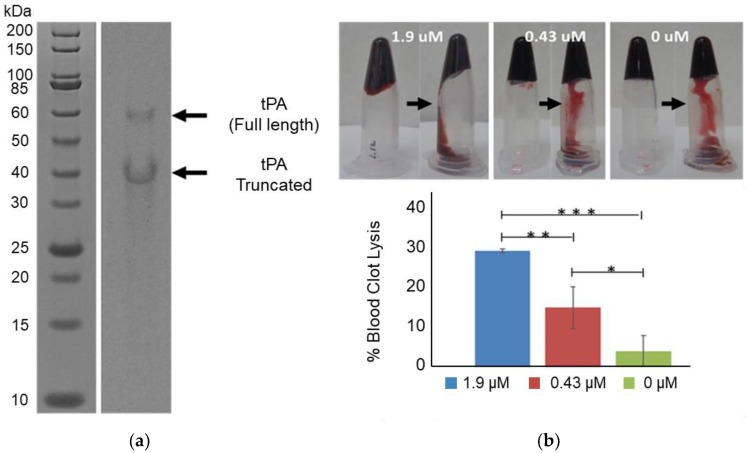
Purification and Activity of tPA. Nickel-affinity column-purified tPA was separated by SDS-PAGE and the gel was exposed to autoradiography film. Samples were also tested with a blood clot lysis assay: (**a**) Autoradiogram analysis showed both a full-length (63 kDa) and truncated tPA (about 40 kDa); (**b**) Analysis of blood clot lysis with CFPS expressed tPA. *** = *p* < 0.005, ** = *p* < 0.05, and * = *p* < 0.1. Error bars represent one standard deviation.

To facilitate the expression and proper folding of tPA, 5 mM of glutathione buffer (with a 4:1 ratio of oxidized and reduced glutathione) was combined with cell extract containing overexpressed GroEL/ES and DsbC in a CFPS reaction (Figure 1a). Combining the above glutathione buffer with DsbC has previously been shown to enable the high yielding production of disulfide-bonded proteins with CFPS [30]. tPA was expressed at 37 °C for 3 h with CFPS supplemented with DsbC to a final DsbC concentration of 3.2 or 6.5 μM. Higher yields were obtained with 6.5 μM of DsbC, reaching 348 μg/mL of soluble tPa (Figure 1b). As reported previously, adding DsbC has been shown to increase both total and soluble protein yields of disulfide-bonded proteins [19]. However, the extent of total yield increase is higher with tPa. One possible explanation could be a higher propensity of the highly disulfide-bonded tPa to catalyze aggregation when incorrectly folded, which may in turn inhibit transcription/translation machinery. Densitometry results indicate that a total of 30% of the expressed tPa was the full-length protein, meaning the actual total soluble tPa yield is 104 μg/mL (Figure 2a). tPa was purified using Ni affinity chromatography and autoradiogram analysis of C14-Leu radiolabeled tPA showed the presence of two protein bands (Figure 2a). Native tPA possesses a total of three glycosylation sites: Asn 117, 184, and 448 [31]. Studies have shown that native tPA is cleaved by serine proteases to form a disulfide-bond linked two-chain tPA when glycosylation is missing at Asn 184 [32,33,34]. Serine proteases present in *E. coli* could cleave non-glycosylated tPA [35] with the distinct truncated band suggesting the product was cleaved by location-specific protease. In the future, this truncation could be reduced by mutagenesis to remove serine protease cleavage sites or engineering of glycosylation into the *E. coli*-based CFPS.

Using the SensoLyte AMC tPA activity assay kit [36], the specific activity of purified tPA was determined to be 3000 μmol/min/mg. This specific activity correlates to less than 1% of the reported specific activity of commercially available tPa [24]. The presence of any activity is encouraging considering the 17 correctly linked disulfide bonds among the 35 cystine residues required for activity, the lack of glycosylation, and the significant concentration of truncated tPa product. However, significant optimization is required before a tPa biosimilar from an *E. coli*-based CFPS system could be realized. This includes engineering of CHO-type glycosylation and tuning the redox potential and chaperone concentrations to better facilitate disulfide bond formation. Engineering glycosylation into *E. coli*-based CFPS appears to be the biggest challenge to the production biosimilars that require glycosylation. Fortunately, recent engineering work engineering asparagine-linked glycosylation in *E. coli*-based CFPS suggests engineering CHO-type glycosylation may become a reality sooner than previously anticipated [37].

### 3.2. Blood Clot Lysis

*E. coli*-based CFPS expressed and purified tPA was also assessed for its ability to lyse human blood clots (Figure 2b). Three samples were prepared with varying tPA concentrations (1.9, 0.43, and 0 μM). Each sample was added to human coagulated blood. After overnight incubation at 37 °C, non-coagulated blood was cleared, and the weight of the remaining blood was measured. About 30% of the coagulated blood was lysed when 1.9 μM tPA was added to the coagulated blood sample. The clot lysis activity of the 1.9 μM tPa samples were statistically greater than the 0.43 μM and 0 μM samples (*p* < 0.05 and *p* < 0.005 respectively). The 0.43 μM tPa samples also appeared to be more active than the control sample with 0 μM tPa with an average of ~15% clot lysis activity compared to an average of ~5%. However, the difference was not as statistically significant (*p* < 0.1). Overall, these results demonstrate that tPa expressed using *E. coli*-based CFPS activated blood clot lysis.

## 4. Conclusions

Here, we demonstrate for the first time the production of active tPA using *E. coli-*based cell-free protein synthesis. Recent advances using low-cost *E. coli*-based CFPS has increased its fitness to produce both biosimilars and new therapeutic proteins. For example, this system can be used to optimize the site of PEGylation which is an important post-translational modification in the development of 2nd-generation drugs [8]. In addition, this system has very recently been used to produce a biosimilar to FDA-approved crisantaspase [21]. However, the system is currently limited to non-glycosylated therapeutics. Continued research engineering glycosylation pathways into the CFPS system is essential to enabling glycosylated biosimilar production in the future.
